# Molecular Imaging of Cell Death in Tumors. Increasing Annexin A5 Size Reduces Contribution of Phosphatidylserine-Targeting Function to Tumor Uptake

**DOI:** 10.1371/journal.pone.0096749

**Published:** 2014-05-06

**Authors:** Lisette Ungethüm, Martijn Chatrou, Dennis Kusters, Leon Schurgers, Chris P. Reutelingsperger

**Affiliations:** Department of Biochemistry, Cardiovascular Research Institute Maastricht, Maastricht University, Maastricht, The Netherlands; West German Cancer Center, Germany

## Abstract

**Objective:**

Annexin A5 is a phosphatidylserine binding protein that binds dying cells *in vivo.* Annexin A5 is a potential molecular imaging agent to determine efficacy of anti-cancer therapy in patients. Its rapid clearance from circulation limits tumor uptake and, hence, its sensitivity. The aim of this study is to determine if non-invasive imaging of cell death in tumors will benefit from increasing circulation time of annexin A5 by increasing its size.

**Procedures:**

Annexin A5 size was increased by complexation of biotinylated annexin A5 with Alexa-Fluor680-labeled streptavidin. The non-binding variant of annexin A5, M1234, was used as negative control. The HT29 colon carcinoma xenograft model in NMRI nude mice was used to measure tumor uptake *in vivo*. Tumor uptake of fluorescent annexin A5-variants was measured using non-invasive optical imaging.

**Results:**

The annexin A5-streptavidin complex (4∶1, moles:moles, M_w_ ∼200 kDa) binds phosphatidylserine-expressing membranes with a Hill-coefficient of 5.7±0.5 for Ca^2+^-binding and an EC50 of 0.9±0.1 mM Ca^2+^ (EC50 is the Ca^2+^ concentration required for half maximal binding)(annexin A5: Hill-coefficient 3.9±0.2, EC50 1.5±0.2 mM Ca^2+^). Circulation half-life of annexin A5-streptavidin is ±21 minutes (circulation half-life of annexin A5 is ±4 min.). Tumor uptake of annexin A5-streptavidin was higher and persisted longer than annexin A5-uptake but depended less on phosphatidylserine binding.

**Conclusion:**

Increasing annexin A5 size prolongs circulation times and increases tumor uptake, but decreases contribution of PS-targeting to tumor uptake and abolishes power to report efficacy of therapy.

## Introduction

Early assessment of efficacy of anticancer drugs can help to improve treatment of cancer patients. Traditionally response to treatment is evaluated by monitoring changes in tumor volume, which often become apparent 4–6 weeks after start of therapy. Molecular Imaging holds the promise to record responses much earlier by unveiling molecular changes at the cellular level [1], [2]. Most anticancer compounds and radiotherapy aim to provoke cell death of tumor cells and endothelial cells of the tumor vasculature. Cell death can present itself in various forms depending on cell death trigger and the subsequently activated pathways [3], [4]. Cell surface expression of phosphatidylserine (PS) appears to be a common denominator to the various modes of cell death and, hence, is an attractive target to measure cell death by Molecular Imaging [5]–[7]. Annexin A5 (anxA5), a PS binding human protein, has been investigated extensively as probe to measure cell death non-invasively [8]–[11]. ^99m^Technetium labeled anxA5 has been used in clinical studies and shows promise in determining efficacy of anticancer therapy early after start of treatment [12]–[16]. The rapid pharmacokinetic of ^99m^Tc-anxA5 [17] is considered a feature that limits tumor uptake and that, thereby, reduces sensitivity of the imaging protocol. AnxA5 has been coupled to polyethylene glycol and nanostructures to prolong circulation time and to improve tumor uptake [18], [19]. Increasing size, however, may diminish contribution of targeting function to uptake by solid tumors due to the enhanced permeability and retention (EPR) effect [20], [21].

In this paper we investigated effects of increasing size of anxA5 on PS-targeting to a tumor in a cyclophosphamide (CYP) treated HT29 human colon carcinoma xenograft mouse model using non-invasive optical imaging.

## Materials and Methods

### Expression, Purification and Labeling of 36 KDa anxA5 Variants

A non-PS binding variant of anxA5 (M1234) was generated by mutation of anxA5 cDNA replacing 4 acidic amino acids of the Ca^2+^ binding sites as described [22]. AnxA5 and M1234 cDNA were cloned into the pQE30-FXa vector (Qiagen) directly behind the factor Xa cleavage site. Gly166Cys and Cys316Ser substitutions were introduced for site-specific maleimide labeling at the concave side of anxA5 [23]. Proteins were expressed by the M15 E. coli strain (Qiagen) and purified from cell lysate using Ni-NTA technology (GE Healthcare). His-tags were removed by FXa cleavage and subsequent Ni-NTA chromatography. Residual FXa was removed on a Benzamidine column (GE Healthcare) and was less than 50 pM for all purified anxA5 variants. Protein purity was verified by SDS-PAGE and MALDI TOF/TOF (Applied Biosystems 4800 analyzer). AnxA5 and M1234 were labeled with maleimide-biotin (Pierce) and maleimide-Alexa Fluor 680 (AF680, Invitrogen/Life Technologies) for 1 hour at room temperature at pH and protein:label molar ratio of 7.4 and 1∶5 (maleimide-biotin) and 6.0 and 1∶1 (maleimide-AF680). Free label was removed by dialysis and labeling efficiency and stoichiometry were assessed by MALDI TOF/TOF analysis. PS binding was measured using ellipsometry as described [24].

### Assembly and Characterization of anxA5-NP and M1234-NP Complexes

Biotinylated anxA5 and M1234 were mixed with streptavidin-AF680 (Invitrogen/Life Technologies) at a molar ratio of 4∶1 and incubated during 30 minutes at room temperature. AnxA5 and M1234 complexed with streptavidin (anxA5–NP and M1234-NP, respectively) were analyzed for PS binding to synthetic phospholipid membranes and apoptotic cells as described elsewhere [25]. The affinity of anxA5-AF680 and anxA5-NP-AF680 binding to PS-membranes was determined using the Red Blood Cell (RBC) assay [26]. Briefly, 1 nM anxA5-AF680 or anxA5-NP-AF680 was incubated with 5*10^7^ RBC/ml (Beckman Coulter) in the presence of calcium concentrations in the range of 0–10 mM. RBC were washed with buffer of equal calcium concentration and bound anxA5-AF680 or anxA5-NP-AF680 was eluted with 600 µl 5 mM EDTA buffer, pH 7.4. Fluorescence at 702 nm (F) was measured in supernatant (SLM Aminco). The binding parameters EC_50_ ([Ca^2+^] required for half-maximal binding) and Hill coefficient (h) were determined from calcium titrations by fitting data to equation F/F_max_ = [Ca]^h^/([Ca]^h^+EC_50_^h^) using GraphPad Prism.

### Relationship between Fluorescence Intensity (FI) and Concentration Fluorescent anxA5 Variant

Dose-ranges of the fluorescent anxA5-variants were prepared in 96-well plates. Fluorescence was measured per well with Optix MX2 (ART Advanced Research Technology, equipped with a 670 nm pulsed laser diode with 80 MHz frequency and 12.5 ns time window) using a 693 nm longpass filter. FI/volume was calculated using Optix MX2 Optiview software (version 2.01.00). Reference curves were prepared by plotting FI/volume versus protein concentration. Fitted reference curves were used to calculate protein concentrations from determined FI/volume in all further experiments.

### HT29 Human Colon Carcinoma Xenograft Mouse Model

Human colon carcinoma HT29 cells (ATCC) were cultured in DMEM supplemented with 10% FBS and 1% penicillin/streptomycin. Cells were harvested with trypsin and resuspended in Phosphate buffered saline, pH 7.4. 15*10^6^ cells were injected subcutaneously into the hind thigh of an immunodeficient NMRI nude mouse (Charles River). Mice having tumor volumes of 200±50 mm^3^ as determined with a caliper, were assigned randomly to control group and treatment group. The treatment group received 170 mg/kg CYP (Sigma-Aldrich) intraperitoneally (IP) 24 hours prior to the start of a series of imaging [27].

### Imaging Tumor-uptake of Fluorescent anxA5 Variants

Mice (n = 7 per group) were anaesthetized with 2.5% isoflurane. Tumor volumes were measured and mice were placed in a holder in the Optix MX2. Region of interest (ROI) was drawn around the tumor and a background image was acquired. 2 nmoles fluorescent anxA5 variant were injected intravenously per mouse and a series of imaging was performed at various time points during a period of one week. FI of ROI was determined using Optix MX2 Optiview software, FI/tumor volume was calculated and subtracted with background signal. Tumor uptake of fluorescent anxA5 variant was calculated in nM from FI/volume using reference curves. Scattering and absorption by tissue was ignored as it was assumed to be comparable for all anxA5 variants.

### Pharmacokinetics of Fluorescent anxA5 Variants

NMRI nude mice (Charles River) were anaesthetized using 2.5% isoflurane and injected with 2 nmoles fluorescent anxA5 variants (n = 3 per group). Blood samples were taken by vena saphena puncture at 1, 5, 15, 30, 60 and 120 minutes and at 1, 15, 30, 60, 120 and 240 minutes post-injection for anxA5 and M1234, and anxA5-NP and M1234-NP, respectively. Blood samples were anticoagulated with 5 mM EDTA. Blood plasma was prepared by centrifugation (5 min in an Eppendorf table top centrifuge) and transferred to wells of a 96-well plate. FI/volume was determined and plasma levels of fluorescent anxA5 variant in nM were calculated. Half-life of circulating anxA5 variant was determined by non-linear regression of the time-course performing a mono-exponential fit.

### Biodistribution of Fluorescent anxA5 Variants

2 nmoles of fluorescent anxA5 variant were administered intravenously to untreated tumor-bearing mice (n = 3 per group). Total body scans were obtained before injection and 1hour (anxA5 and M1234) and 24 hours post-injection (anxA5-NP and M1234-NP) using the Optix MX2 system with a scan resolution of 1 mm. Hereafter mice were sacrificed, organs were collected, weighted and FI was measured per organ. FI/volume was calculated assuming that all organs had a density of 1 g/ml. Concentrations of variants were calculated in nM using reference curves.

### Circulating PS-expressing Microparticles (MPs)

Blood was collected from non-tumor bearing mice and from tumor-bearing mice before and 24 hours after treatment with CYP (n = 5 per group) via cardiac puncture. Blood was anticoagulated with citrate and amount of PS-expressing MPs was determined using the Zymuphen MP-activity kit protocol (Hyphen Biomed).

### Immunohistochemical Analysis of Cell Death and Microvessel Density (MVD)

Frozen tumors were cut into 7 µm thick sections and fixed with 4% paraformaldehyde. Cell death was visualized using the TdT-mediated dUTP-X nick end labeling (TUNEL) assay according to the manufacturer’s protocol (In situ cell death detection kit, POD –Roche Applied Science). Nuclei were counterstained with hematoxylin (Klinipath). 4 Images of 2 sections of each tumor were collected at 20 × magnification (Leica DM 2000). MVD was determined using rat anti-mouse CD31 mAb and rabbit anti-rat IgG (BD Pharmingen), Brightvision poly HRP anti-rabbit IgG (Immunologic) and NovaRed-staining (Vector Labs). TUNEL-positivity and MVD-density were expressed as % TUNEL positivity per nuclear area, and CD31-staining per total area using ImageJ [28] with the Immunoratio plugin [29].

### Tumor Compartmental Modeling

A 3-compartmental model adapted from Bartlett et al. [30] was used to investigate the influence of active tumor targeting on tumor accumulation and retention of anxA5 variants. The system of equations was solved using Mathematica software (Wolfram Research).

### Statistics

A two-tailed Student’s t-test was used to determine significance unless stated otherwise. Differences were considered significant for p<0.05 (*), p<0.01 (**) and p<0.001 (***).

### Ethical Statement

All experiments with animals were approved by the local Animal Experiment Committee of the Maastricht University (DEC-UM).

## Results

### Characterization of Fluorescent anxA5, M1234, anxA5-NP and M1234-NP

**Figure 1:**
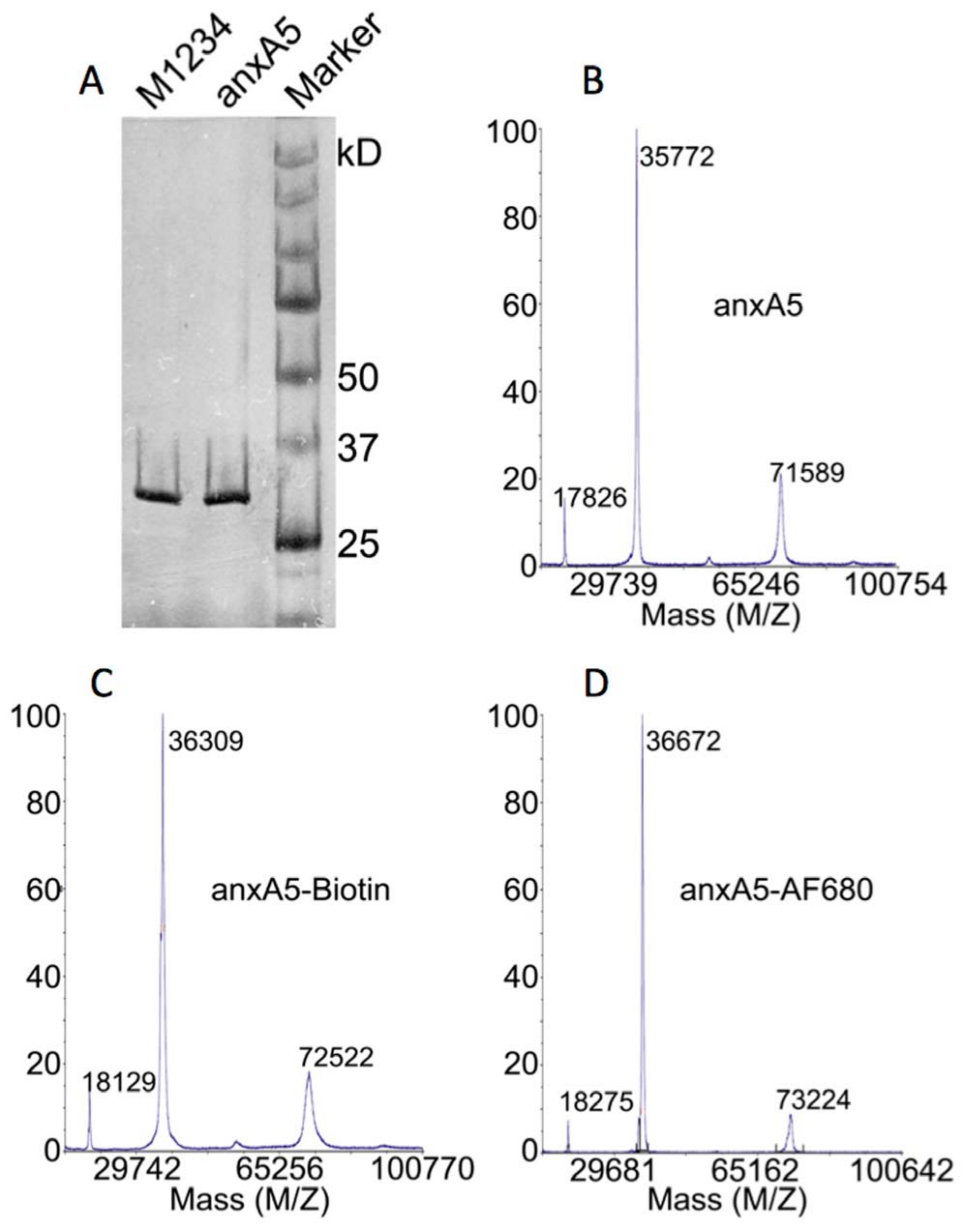
Analyses of purity of anxA5 and M1234, and stoichiometry of anxA5-biotin and anxA5-AF680. (A) 50 ng purified anxA5 and M1234 were run on an SDS-PAGE. The proteins were visualized by silver staining. (B) Maldi TOF/TOF spectrum of purified anxA5 showing a peak of the monomer at 35772 Da, the dimer at 71589 Da and the double protonated peak at 17826 Da. (C) Maldi TOF/TOF spectrum of anxA5 labeled with maleimide-biotin showing a peak at 36309 Da. Maleimide-biotin labeling resulted in a Mw increase of ±540 Da indicating 1∶1 stoichiometry. (D) Maldi TOF/TOF spectrum of anxA5 labeled with maleimide-AF680 showing a peak at 36672 Da. Maleimide-AF680 labeling resulted in a Mw increase of ±900 Da indicating 1∶1 stoichiometry. Comparable results were obtained with M1234-biotin and M1234-AF680 (not shown).

**Figure 2:**
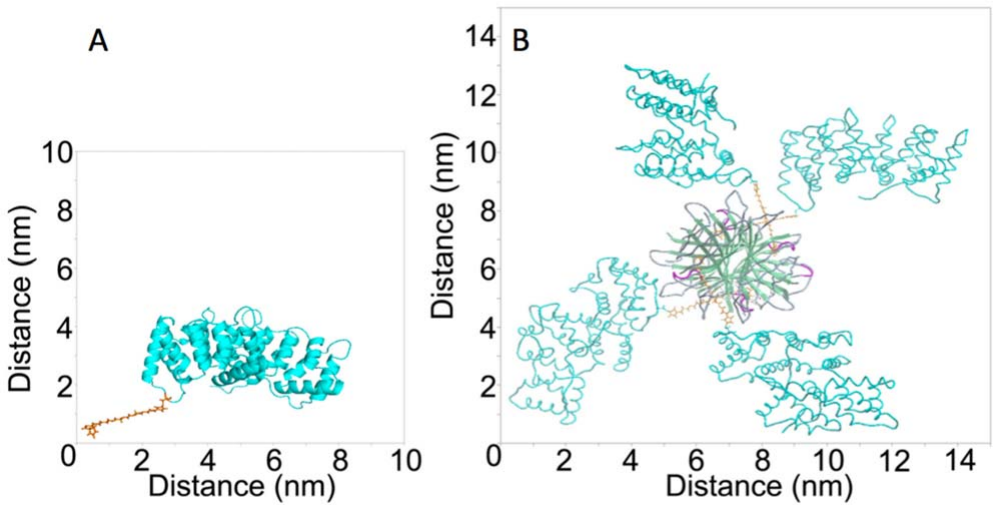
Three-dimensional (3-D) models of anxA5-Biotin (A) and anxA5-NP (B). The 3-D structure was retrieved from the 1ANX entry of the Protein Data Bank (PDB). Residue 166 was replaced by a cysteine and coupled to maleimide-PEG2-Biotin using Yasara. The picture was generated using PyMOL. The structure of streptavidin was retrieved from the 1SWG entry of PDB. 4 anxA5-biotin monomers were docked to streptavidin’s biotin binding pockets and the picture was generated using ICM-Pro.

**Figure 3:**
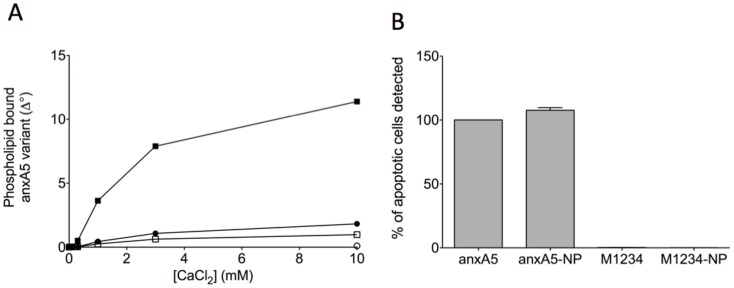
Binding of annexin-variants to phosphatidylserine containing membranes. Panel A shows calcium-dependent binding of anxA5 (closed circles), M1234 (open circle), anxA5-NP (closed squares) and M1234-NP (open squares) to a synthetic phospholipid surface comprising 20 mole% phosphatidylserine and 80 mole% phosphatidylcholine. Binding was measured by ellipsometry and is expressed as change in degree of the analyser (Δ°) as described elsewhere [24]. Panel B shows the % of apoptotic cells of a population of anti-Fas stimulated Jurkat cells that can be detected using fluorescently labeled annexin-variants and flow cytometry.

G166C variants of anxA5 and M1234 labeled with maleimide-AF680 and maleimide-biotin showed 1∶1 stoichiometric complexes (Figure 1). The anxA5-NP and M1234-NP are fluorescent nanoparticles of ∼200 kDa with estimated dimensions of approximately 14×14×8 nm (Figure 2). Conjugation to Cysteine at position 166 should be without effect on the functional PS-binding of anxA5 even if large molecular complexes were conjugated [31]. AnxA5 and anxA5-NP bound Ca^2+^-dependently to PS-containing phospholipid bilayers (Figure 3A) and to apoptotic cells (Figure 3B) whereas both M1234, a non-PS binding variant of anxA5 [23], and M1234-NP had greatly reduced PS-binding activity (Figure 3).

**Figure 4:**
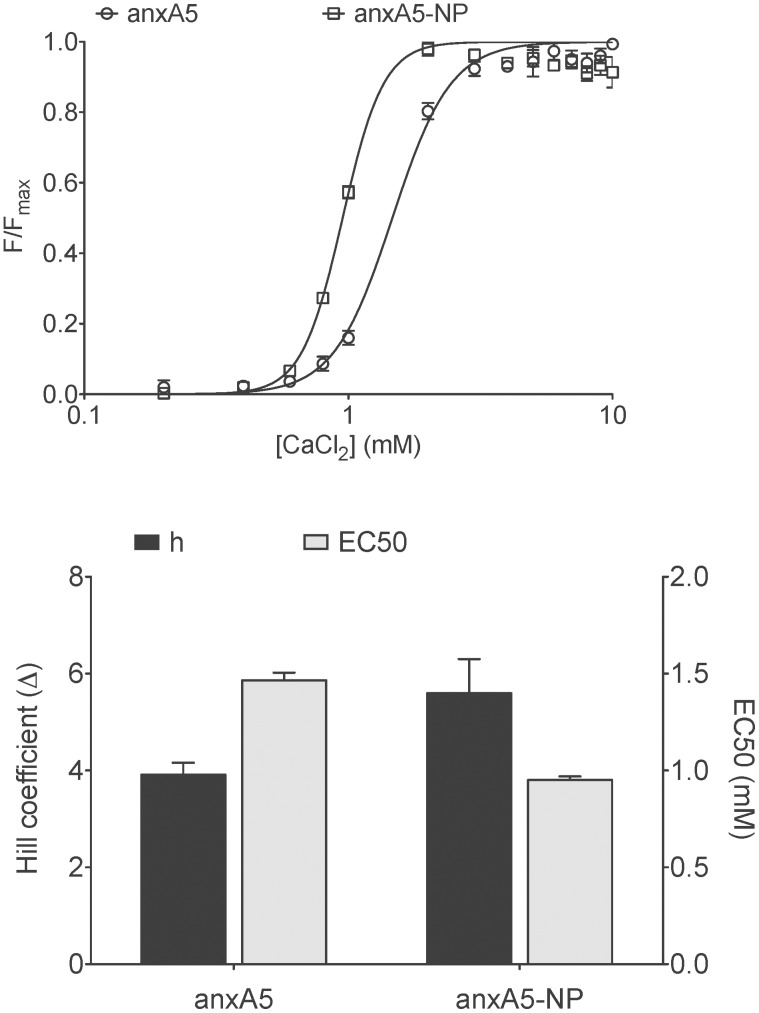
Red blood Cell (RBC) binding of anxA5 variants at low membrane density (1*10^4^ molecules/cell) as determined by calcium titration. (A) Calcium titration curves of anxA5 (open circles) and anxA5-NP (open squares). Each point is the average of three experiments with bars indicating SEM. (B) Hill coefficient (black bars) and EC50 (grey bars) of anxA5 and anxA5-NP as determined from calcium titration curves.

PS-binding affinity of anxA5 and anxA5-NP were measured using the RBC-assay. AnxA5 and anxA5-NP had Hill-coefficients for Ca^2+^-binding of 3.9±0.2 and 5.7±0.5 respectively, and EC50-values (Ca^2+^-requirement for half-maximal binding to the RBC surface) of 1.5±0.2 mM and 0.9±0.1 mM repectively (Figure 4), demonstrating that anxA5-NP exhibits a higher affinity for PS-expressing cell membranes at extracellular Ca^2+^-levels than anxA5. Relationships between fluorescent anxA5 variant concentration and Fluorescence Intensity (FI) were linear for all anxA5-variants (data not shown).

### Pharmacokinetics and Biodistribution of anxA5, M1234, anxA5-NP and M1234-NP

**Figure 5:**
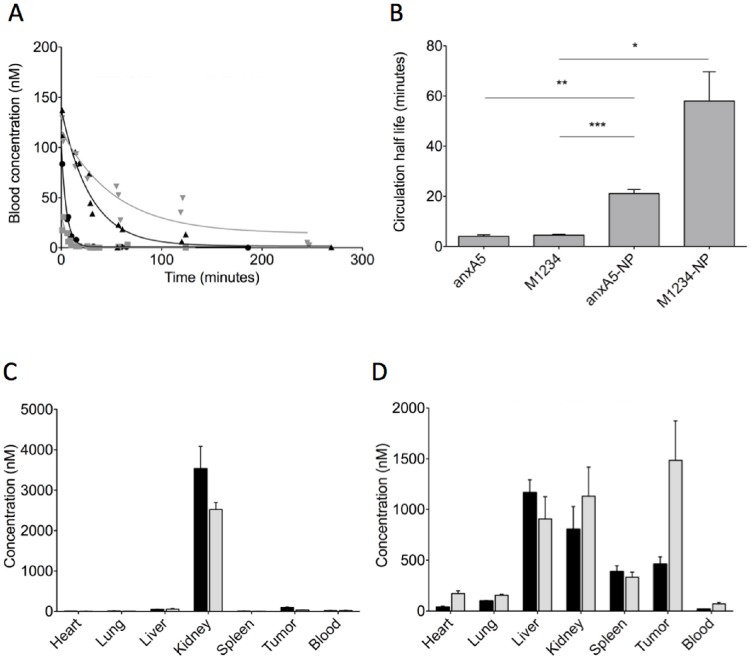
Blood clearance and biodistribution of annexin-variants in NMRI nude mice. 2-labeled annexin-variant were injected intravenously. At various time-points 25 µl blood samples were collected to measure fluorescence. Blood concentration of annexin-variant was calculated using the constructed reference curves. Panel A shows the blood clearance of anxA5 (grey squares), M1234 (black circles), anxA5-NP (black triangles) and M1234-NP (grey triangles). Half-lives were calculated by mono-exponential fit (B). *p<0.05, **p<0.01, ***p<0.001. 2 nmoles of fluorescently labeled anxA5 (black bars, panel C), M1234 (grey bars, panel C), anxA5-NP (black bars, panel D) and M1234-NP (grey bars, panel D) were injected intravenously into tumor-bearing NMRI nude mice. 1 hour (panel C) and 24 hours (panel D) post-injection organs were collected, weighed and measured for fluorescence. Concentration of annexin-variant were calculated using the constructed reference curves.

Pharmacokinetics of the fluorescent anxA5-variants were determined by injecting 2 nmoles of fluorescent anxA5-variant intravenously into NMRI nude mice. All fluorescent conjugates showed mono-exponential clearance from circulation (Figure 5A) from which half-lives were calculated of 4.1, 4.5, 21 and 58 minutes for anxA5, M1234, anxA5-NP and M1234-NP, respectively (Figure 5B). These findings suggest that the property of PS-binding causes a more rapid clearance from circulation. Circulating microparticles (MP) with surface expressed PS may explain this observation [32]. Therefore, we determined PS-expressing MPs in circulation of control mice and untreated and CYP-treated tumor-bearing mice. Levels of circulating PS-expressing MPs were in all cases below 0.5 nM of PS-equivalents and were too low to cause significant sequestration of injected anxA5 and anxA5-NP (data not shown). Biodistributions of anxA5, M1234, anxA5-NP en M1234-NP were determined in tumor-bearing mice. In contrast to anxA5 and M1234, which are predominantly cleared by kidneys (Figure 5C), anxA5-NP and M1234-NP showed higher liver and spleen uptake in agreement with size of the NP (Figure 5D). AnxA5 accumulated more in the tumor than M1234 (Figure 5C) while this was reversed for anxA5-NP and M1234-NP (Figure 5D).

### Optical Imaging of Response to CYP-treatment using Fluorescent anxA5-variants

**Figure 6:**
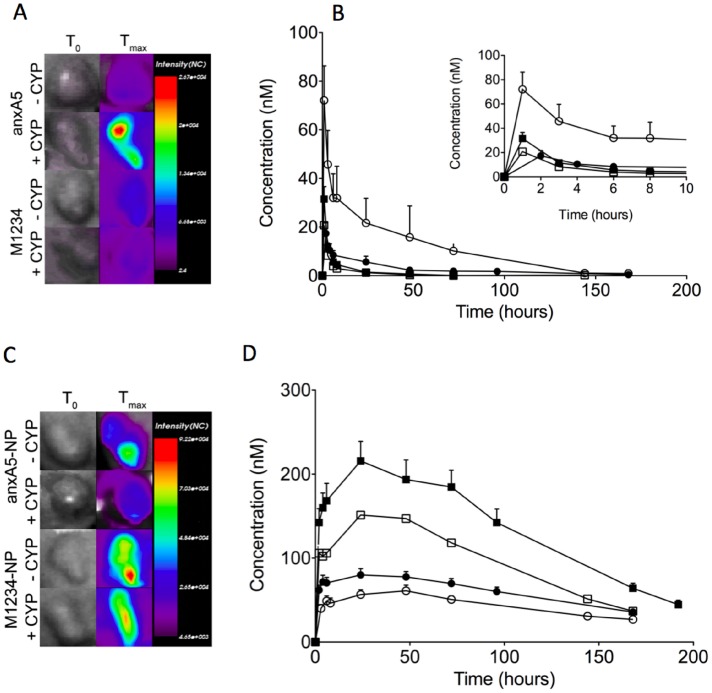
Non-invasive optical imaging of kinetics of tumor-uptake of fluorescently labeled annexin-variants in tumor bearing NMRI nude mice untreated (–CYP) or treated with cyclophosphamide (+CYP). Panels A and C show representative pictures of tumors of mice injected with anxA5 and M1234 (A), and anxA5-NP and M1234-NP (C). Panel B illustrates the time courses of tumor levels of anxA5 (closed circles, –CYP; open circles, +CYP) and M1234 (closed squares, –CYP; open squares, +CYP). The inset shows the time-courses during the first 10 hours. Panel D represents the time-courses of tumor levels of anxA5-NP (closed circles, –CYP; open circles, +CYP) and M1234 (closed squares, –CYP; open squares, +CYP).

**Figure 7:**
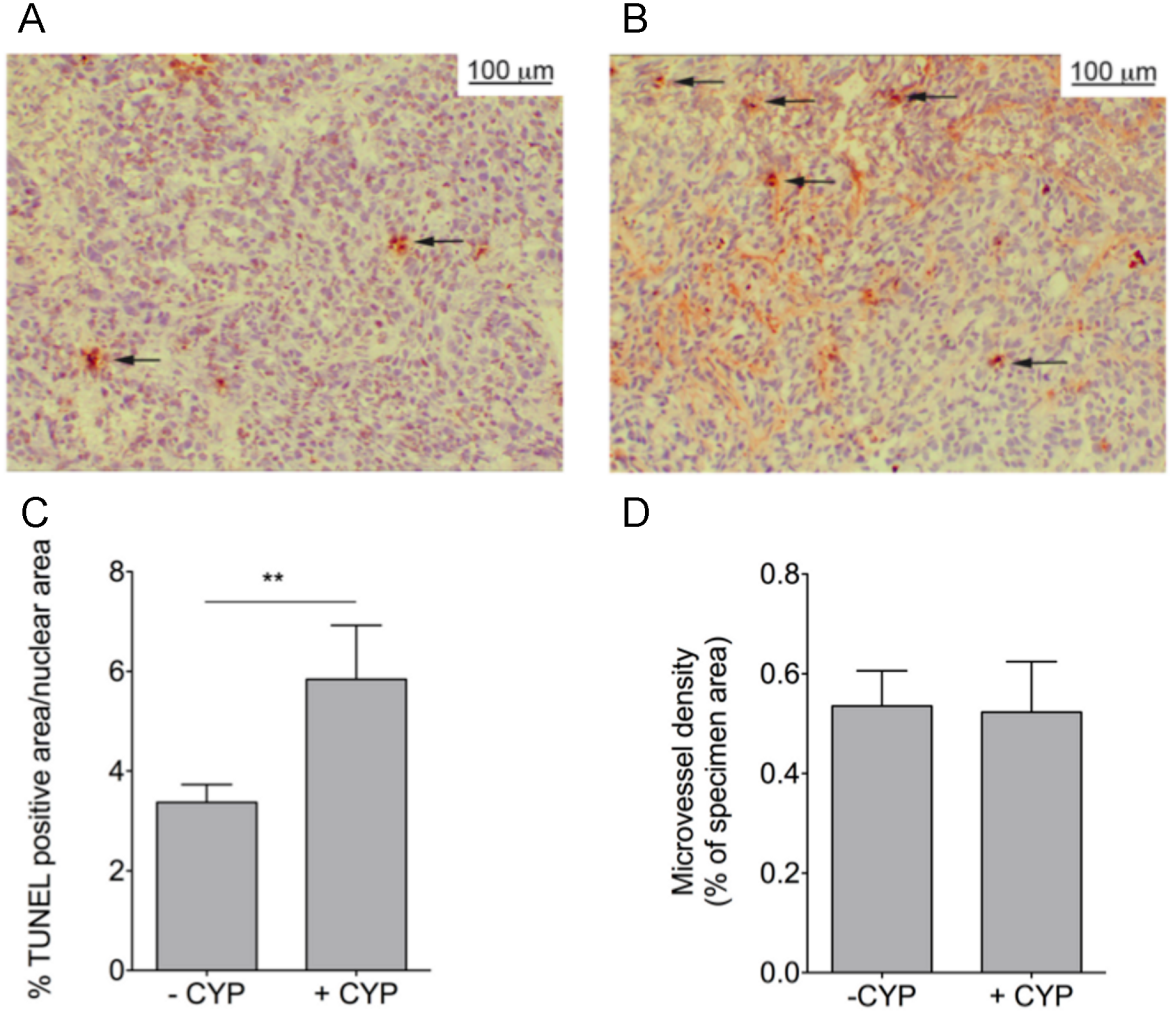
Determination of apoptosis (A–C) and microvessel density (D) in tumors of NMRI nude mice: effects of cyclophosphamide (CYP) treatment. Untreated (–CYP) and treated tumors (+CYP) were excised from tumor-bearing mice, frozen and sectioned. Sections were stained with TUNEL-assay and hematoxylin. % TUNEL-positive nuclei was determined as a measure of apoptosis (A–C). Panel A shows TUNEL-staining of a section of an untreated tumor and panel B of a CYP-treated tumor. Sections were stained with the endothelial specific antibody anti-CD31 and hematoxylin. Microvessel density was determined as % of tumor area that is CD31-positive (D). **p<0.01.

Biodistribution was determined at a single time-point post-injection of the variants. In order to determine contribution of size and PS-targeting to tumor uptake of the variants we measured dynamics of tumor uptake in untreated and CYP-treated tumor-bearing mice by non-invasive optical imaging during a period upto 200 hrs (Figure 6). AnxA5 accumulated more than M1234 and its tumor level increased by CYP-treatment during the time-course in contrast to M1234, the uptake of which showed a decrease in response to CYP-treatment (Figure 6B). AnxA5-NP and M1234-NP also accumulated in tumor, albeit with kinetics and at levels different from those of the monomers (Figure 6D). Remarkably, M1234-NP showed higher uptake than anxA5-NP during the whole time-course even if the tumor was treated with cyclophosphamide. CYP-treatment significantly reduced tumor uptake of both NP’s (Figure 6D). In order to gain understanding at the cellular level for the CYP-induced differences in tumor uptake we measured both cell death and microvessel density (MVD). CYP-treatment caused an increase of cell death in the tumor (Figure 7A, B) explaining the higher uptake of anxA5 by CYP-treated tumor. CYP-treatment was without effect on MVD (Figure 7C, D).

### Compartmentalization of Fluorescent anxA5-variants in Untreated and CYP-treated Tumor

**Figure 8:**
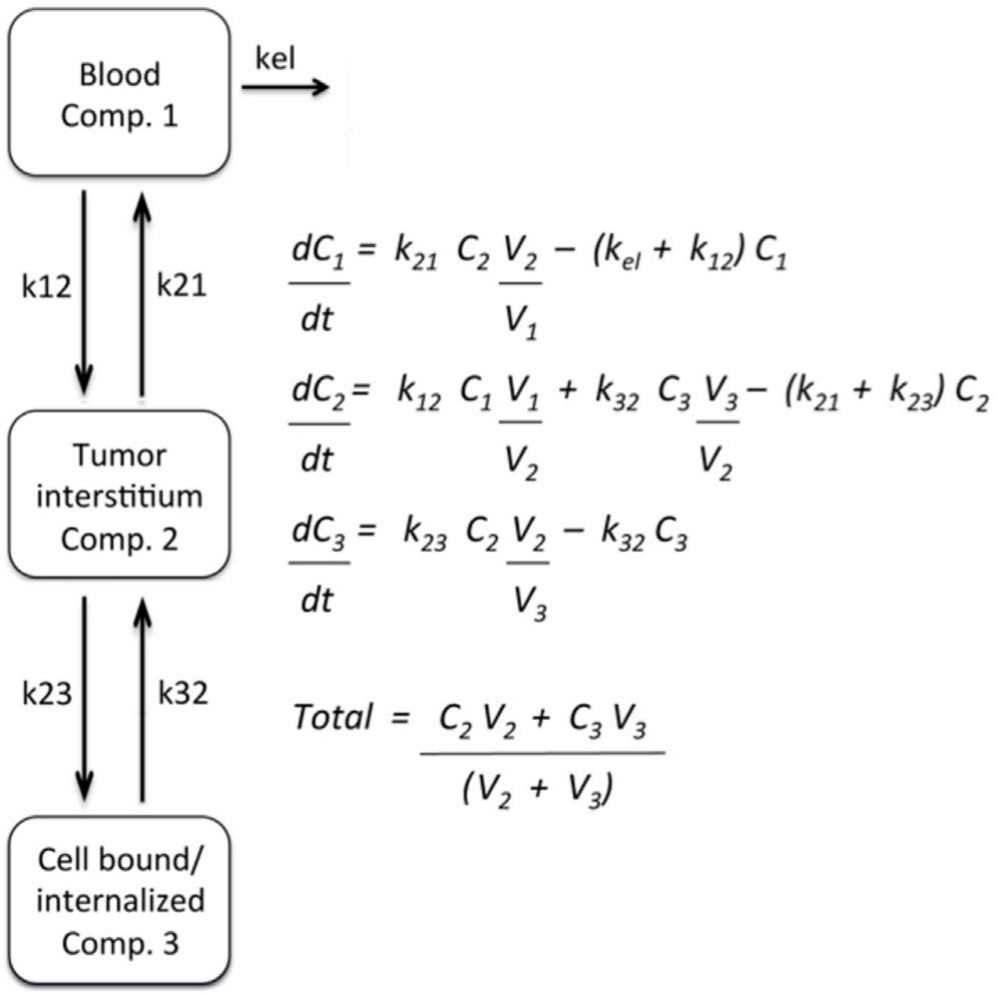
3-compartment model and equations that were employed to fit the experimental data. k is constant, C is concentration (nM) and V is volume (L).

**Figure 9:**
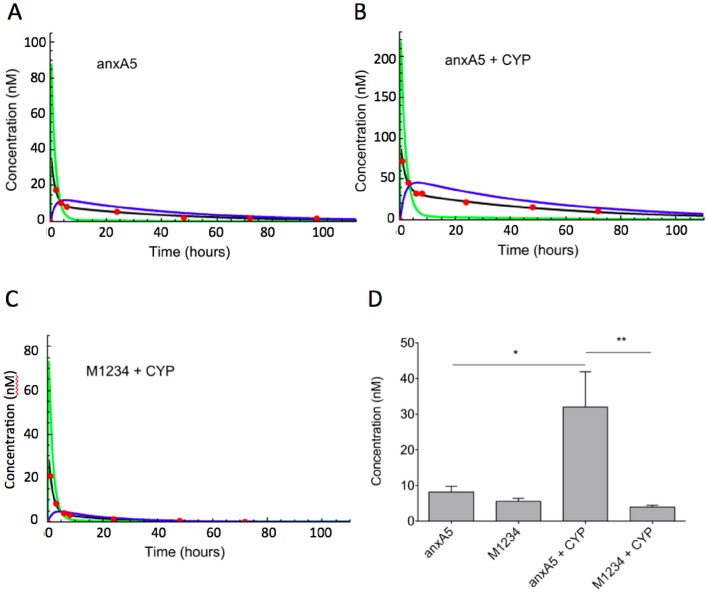
Computational compartmentalization of tumor uptake of anxA5 and M1234. The experimental data of Fig. 6B were fit according to the model of Fig. 8. Panels A–C show representative fits for anxA5 uptake by untreated (A) and cyclophosphamide treated tumor (B) and for M1234 uptake by cyclophosphamide treated tumor (C.) The experimental data (red circles) were used to calculate concentrations in whole tumor (black line), tumor interstitium (Comp. 2, red line) and bound to the PS-target (Comp. 3, blue line). Panel D shows the concentrations of anxA5 and M1234 in compartment 3 (PS-target) of tumor that was either untreated or treated with cyclophosphamide (+CYP) 6 hours post-injection. *p<0.05, **p<0.01.

In order to assess dynamics of distribution of variants within the tumor we adopted a non-linear bi-phasic 3-compartment model assuming exchange between compartment 1 (blood) and compartment 2 (tumor interstitium) and between compartment 2 and compartment 3 (PS-target) (Figure 8). The experimental data of Figure 6B and not those of Figure 6D could be fitted according to the 3 compartment model. On basis of the fit the total uptake of anxA5 and M1234 could be dissected into dynamics of uptake in compartment 2 and 3 (Figure 9). AnxA5 reached peak concentrations in the PS-target compartment approximately 6 hours post-injection (Figure 9A). Peak concentration of anxA5 in the PS-target compartment was increased approximately 4-fold by CYP-treatment (Figure 9B, D). M1234 concentration on the other hand peaked around 4 hours and decreased in response to CYP-treatment (Figure 9C, D).

## Discussion

Molecular Imaging holds promise to predict efficacy of anti-cancer treatment of patients early after start of therapy. Several tumor targets and imaging modalities potentially serve this purpose [2]. Cell death of a tumor is regarded amongst the most promising targets and anxA5, which binds dying and dead cells, is appreciated as the most promising Molecular Imaging-agent. Radiolabeled anxA5 has been evaluated in clinical studies [8] and the clinical experience, although promising, indicate that the anxA5 imaging protocol requires improvement in order to be applicable to a broad range of cancer types and treatment regimens [16]. Timing of imaging is one of the critical parts of the protocol because the cell death response of the tumor may occur between 1 and 48 hours after start of treatment depending on cancer type and treatment regimen and because anxA5 is rapidly cleared from the circulating blood [33], [34]. A protocol of multiple injections with labeled anxA5 was proposed to deal with the critical issue of timing [35]. Another proposed strategy aims to prolong circulation time by increasing size of anxA5 so as to circumvent need for multiple injections [19]. But size is key to tumor uptake and retention by determining passage of the compound across vascular endothelium, interstitial diffusivity and lymphatic drainage, collectively called the Enhanced Permeability and Retention (EPR) effect. EPR is a general characteristic of vascularized solid tumor [21] and becomes effective if compounds are larger than 40 kDa [36]. Increasing size of anxA5 above this threshold would, hence, change maximal tumor uptake, time-course of uptake and contribution of target affinity to uptake [37]. In order to study effect of increasing size of anxA5 on dynamics of tumor uptake we conducted non-invasive molecular imaging using the well-vascularized HT29 tumor model in NMRI nude mice. Since HT29 tumors can be treated effectively with CYP [38] we used this cytotoxic agent to increase PS-target in tumors. Firstly, we measured tumor-uptake of anxA5 and M1234 by non-invasive imaging. AnxA5 uptake exceeded always M1234 uptake indicating that PS-binding contributed to anxA5 accumulation in the tumor. This was underscored by the observation that CYP-treatment increased apoptosis and anxA5 uptake but not M1234 uptake. Interestingly, CYP-treatment reduced M1234 uptake without affecting microvessel density, suggesting that this cytotoxic agent normalizes leaky tumor vessels. This interpretation was strengthened by the observation that CYP-treatment reduces tumor-levels of anxA5-NP and M1234-NP, the uptakes of which are governed by EPR effects (see below). The time-courses of tumor uptake of both anxA5 and M1234 could be fitted almost perfectly with a 3-compartment model [36] assuming exchange between blood (compartment 1) and tumor interstitium (compartment 2) and between tumor interstitium and tumor PS-target (compartment 3). According to this model anxA5-levels in the PS-compartment peaked approximately 6 hours post-injection and increased ±4-fold after CYP-treatment. Hence, the optimal time-point for tumor-imaging would be around 6 hours after injection of labeled anxA5. Others reported optimal imaging time-points between 4 and 6 hours post-injection based on tumor-blood ratios of labeled anxA5 [27], [39], [40].

In order to prolong circulation time and, consequently, tumor uptake, the size of anxA5 was increased by biotinylation and subsequently complexation with streptavidin. This yielded a PS-binding complex of ∼200 kDa with increased circulation half-life and an expected shift of the biodistribution profile from kidneys to liver and spleen. M1234-NP had a longer circulation half-life than anxA5-NP indicating that PS-binding of anxA5-NP contributes to its clearance likely through PS-dependent clearance by the reticuloendothelial system [19], [41], this in contrast to anxA5, the clearance of which is dominated by kidney filtration and less by PS-binding affinity [42]. The kinetics of tumor-uptake of AnxA5-NP and M1234-NP markedly differed from those of the monomers. Tumor-levels of the complexes increased in the first 25 hours after administration, whereafter they gradually declined. The time-courses of tumor-levels of the complexes could not be fitted according to the model of Figure 8, indicating that dynamics of exchange between the compartments differed between complexes and monomers, likely as a result of EPR effects acting on the complexes. Longer circulation times and EPR effects were likely responsible for the higher tumor-levels of the complexes. Remarkably tumor-levels of M1234-NP were always higher than anxA5-NP levels even after treatment with CYP indicating that PS-targeting does not contribute significantly to tumor uptake of anxA5-NP indicating that EPR effects govern uptake and retention of the anxA5-NP. Treatment of the tumor with CYP reduced uptake of both anxA5-NP and M1234-NP suggesting that CYP-treatment reduced EPR. Such phenomena may have consequences for therapeutic strategies combining cytostatics such as CYP and nanoparticles [43].

Altogether our results demonstrate that increasing size of anxA5 to ±200 kDa reduces contribution of PS-binding to uptake in the HT29-tumor model due to EPR effects. Since EPR is an effect that occurs in vascularized solid tumors [44] we expect that increasing size of anxA5 diminishes its suitability as molecular imaging agent to report efficacy of anti-cancer treatment also in other solid tumors. Since EPR effects occur for compounds larger than 40 kDa [36] anxA5 complexes of sizes above this threshold may be less suitable for Molecular Imaging protocols to measure cell death in tumors. However, we have increased size by using streptavidin and biotinylated anxA5 and cannot rule out that increasing size by other methods yielding complexes with different size, shape and surface charge have different relative contributions of PS-targeting to the overall uptake by the tumor.

## References

[1] Weissleder R, Pittet MJ (2008) Imaging in the era of molecular oncology. Nature 452: 580–589. 18385732 10.1038/nature06917 PMC2708079

[2] Michalski MH, Chen X (2011) Molecular imaging in cancer treatment. Eur J Nucl Med Mol Imaging 38: 358–377. 20661557 10.1007/s00259-010-1569-z PMC3022114

[3] Galluzzi L, Vitale I, Abrams JM, Alnemri ES, Baehrecke EH, et al (2012) Molecular definitions of cell death subroutines: recommendations of the Nomenclature Committee on Cell Death 2012. Cell Death Differ 19: 107–120. 21760595 10.1038/cdd.2011.96 PMC3252826

[4] Green DR, Victor B (2012) The pantheon of the fallen: why are there so many forms of cell death? Trends Cell Biol 22: 555–556 doi 22995729 10.1016/j.tcb.2012.08.008 PMC3568685

[5] Corsten MF, Hofstra L, Narula J, Reutelingsperger CP (2006) Counting heads in the war against cancer: defining the role of annexin A5 imaging in cancer treatment and surveillance. Cancer Res 66: 1255–1260. 16452175 10.1158/0008-5472.CAN-05-3000

[6] Vermes I, Haanen C, Steffens-Nakken H, Reutelingsperger C (1995) A novel assay for apoptosis. Flow cytometric detection of phosphatidylserine expression on early apoptotic cells using fluorescein labelled Annexin V. J Immunol Methods 184: 39–51. 7622868 10.1016/0022-1759(95)00072-i

[7] Schutters K, Reutelingsperger C (2010) Phosphatidylserine targeting for diagnosis and treatment of human diseases. Apoptosis 15: 1072–1082. 20440562 10.1007/s10495-010-0503-y PMC2929432

[8] Boersma HH, Kietselaer BL, Stolk LM, Bennaghmouch A, Hofstra L, et al (2005) Past, present, and future of annexin A5: from protein discovery to clinical applications. J Nucl Med 46: 2035–2050. 16330568

[9] Tait JF (2008) Imaging of apoptosis. J Nucl Med 49: 1573–1576. 18794267 10.2967/jnumed.108.052803

[10] Blankenberg FG (2008) In vivo detection of apoptosis. J Nucl Med 49 Suppl 2 81S–95S. 18523067 10.2967/jnumed.107.045898

[11] Kusters DH, Tegtmeier J, Schurgers LJ, Reutelingsperger CP (2012) Molecular imaging to identify the vulnerable plaque-from basic research to clinical practice. Mol Imaging Biol 14: 523–533. 22983911 10.1007/s11307-012-0586-7

[12] Belhocine TZ, Blankenberg FG (2006) The imaging of apoptosis with the radiolabelled annexin A5: a new tool in translational research. Curr Clin Pharmacol 1: 129–137. 18666365 10.2174/157488406776872541

[13] Kartachova MS, Valdes Olmos RA, Haas RL, Hoebers FJP, van Herk M, et al (2008) 99mTc-HYNIC-rh-annexin-V scintigraphy: visual and quantitative evaluation of early treatment-induced apoptosis to predict treatment outcome. Nucl Med Commun 29: 39–44. 18049096 10.1097/MNM.0b013e3282f1bc22

[14] Rottey S, Slegers G, Van Belle S, Goethals I, Van de Wiele C (2006) Sequential 99mTc-Hydrazinonicotinamide-Annexin V Imaging for Predicting Response to Chemotherapy. J Nucl Med 47: 1813–1818. 17079815

[15] Hoebers FJ, Kartachova M, de Bois J, van den Brekel MWM, van Tinteren H, et al (2008) (99m)Tc Hynic-rh-Annexin V scintigraphy for in vivo imaging of apoptosis in patients with head and neck cancer treated with chemoradiotherapy. Eur J Nucl Med Mol Imaging 35: 509–518. 17994297 10.1007/s00259-007-0624-x PMC2275773

[16] Schaper F, Reutelingsperger C (2013) 99mTc-HYNIC-Annexin A5 in Oncology: Evaluating Efficacy of Anti-Cancer Therapies. Cancers 5: 550–568. 24216991 10.3390/cancers5020550 PMC3730331

[17] Kemerink GJ, Liu X, Kieffer D, Ceyssens S, Mortelmans L, et al (2003) Safety, biodistribution, and dosimetry of 99mTc-HYNIC-annexin V, a novel human recombinant annexin V for human application. J Nucl Med 44: 947–952. 12791824

[18] Ke S, Wen X, Wu QP, Wallace S, Charnsangavej C, et al (2004) Imaging taxane-induced tumor apoptosis using PEGylated, 111In-labeled annexin V. J Nucl Med. 45: 108–115. 14734682

[19] Zhang R, Lu W, Wen X, Huang M, Zhou M, et al (2011) Annexin A5-conjugated polymeric micelles for dual SPECT and optical detection of apoptosis. J Nucl Med 52: 958–964. 21571801 10.2967/jnumed.110.083220 PMC3463236

[20] Jain RK, Stylianopoulos T (2010) Delivering nanomedicine to solid tumors. Nat Rev Clin Oncol 7: 653–664. 20838415 10.1038/nrclinonc.2010.139 PMC3065247

[21] Maeda H, Wu J, Sawa T, Matsumura Y, Hori K (2000) Tumor vascular permeability and the EPR effect in macromolecular therapeutics: a review. J Control Release 65: 271–284. 10699287 10.1016/s0168-3659(99)00248-5

[22] Mira JP, Dubois T, Oudinet JP, Lukowski S, Russo-Marie F, et al (1997) Inhibition of cytosolic phospholipase A2 by annexin V in differentiated permeabilized HL-60 cells. Evidence of crucial importance of domain I type II Ca2+-binding site in the mechanism of inhibition. J Biol Chem 272: 10474–10482. 9099690 10.1074/jbc.272.16.10474

[23] Schutters K, Kusters DH, Chatrou ML, Montero-Melendez T, Donners M, et al (2013) Cell surface-expressed phosphatidylserine as therapeutic target to enhance phagocytosis of apoptotic cells. Cell Death Differ 20: 49–56. 22955945 10.1038/cdd.2012.107 PMC3524641

[24] Andree HA, Reutelingsperger CP, Hauptmann R, Hauptmann R, Hemker HC, et al (1990) Binding of vascular anticoagulant alpha (VAC alpha) to planar phospholipid bilayers. J Biol Chem 265: 4923–4928. 2138622

[25] Ungethum L, Kenis H, Nicolaes GA, Autin L, Stoilova-McPhie S, et al (2011) Engineered annexin A5 variants with impaired cell entry for molecular imaging of apoptosis using pretargeting strategies. J Biol Chem 286: 1903–1910. 21078669 10.1074/jbc.M110.163527 PMC3023486

[26] Tait JF, Gibson DF, Schmidt C (2004) Measurement of the affinity and cooperativity of annexin V-membrane binding under conitions of low membrane occupancy. Anal Biochem 329: 112–119. 15136173 10.1016/j.ab.2004.02.043

[27] Takei T, Kuge Y, Zhao S, Sato M, Strauss HW, et al (2004) Time course of apoptotic tumor response after a single dose of chemotherapy: comparison with 99mTc-annexin V uptake and histologic findings in an experimental model. J Nucl Med 45: 2083–2087. 15585485

[28] Schneider CA, Rasband WS, Eliceiri KW (2012) NIH Image to ImageJ: 25 years of image analysis. Nat Meth 9: 671–675. 10.1038/nmeth.2089 PMC5554542 22930834

[29] Tuominen VJ, Ruotoistenmaki S, Viitanen A, Jumppanen M, Isola J (2010) ImmunoRatio: a publicly available web application for quantitative image analysis of estrogen receptor (ER), progesterone receptor (PR), and Ki-67. Breast Cancer Res 12: R56. 20663194 10.1186/bcr2615 PMC2949645

[30] Bartlett D, Su H, Hildebrandt I, Weber WA, Davis ME (2007) Impact of tumor-specific targeting on thebiodistribution and efficacy of siRNA nanoparticlesmeasured by multimodality in vivo imaging. Proc Natl Acad Sci U S A 104: 15549–15554. 17875985 10.1073/pnas.0707461104 PMC1978218

[31] Schellenberger E, Schnorr J, Reutelingsperger C, Ungethüm L, Meyer W, et al (2008) Linking proteins with anionic nanoparticles via protamine: ultrasmall protein-coupled probes for magnetic resonance imaging of apoptosis. Small 4: 225–230. 18203233 10.1002/smll.200700847

[32] Davila M, Amirkhosravi A, Coll E, Desai H, Robles L, et al (2008) Tissue factor-bearing microparticles derived from tumor cells: impact on coagulation activation. J Thromb Haemost 6: 1517–1524. 18433463 10.1111/j.1538-7836.2008.02987.x

[33] Blankenberg FG, Katsikis PD, Tait JF, Davis E, Naumovski L, et al (1998) In vivo detection and imaging of phosphatidylserine expression during programmed cell death. Proc Natl Acad Sci USA 95: 6349–6354. 9600968 10.1073/pnas.95.11.6349 PMC27696

[34] Mandl SJ, Mari C, Edinger M, Negrin RS, Tait JF, et al (2004) Multi-modality imaging identifies key times for annexin V imaging as an early predictor of therapeutic outcome. Mol Imaging 3: 1–8. 15142407 10.1162/15353500200403157

[35] Blankenberg F (2002) To Scan or Not To Scan, It Is a Question of Timing: Technetium-99m-Annexin V Radionuclide Imaging Assessment of Treatment Efficacy after One Course of Chemotherapy. Clin Cancer Research 8: 2757–2758. 12231512

[36] Maeda H, Nakamura H, Fang J (2013) The EPR effect for macromolecular drug delivery to solid tumors: Improvement of tumor uptake, lowering of systemic toxicity, and distinct tumor imaging in vivo. Adv Drug Deliv Rev 65: 71–79. 23088862 10.1016/j.addr.2012.10.002

[37] Schmidt MM, Wittrup KD (2009) A modeling analysis of the effects of molecular size and binding affinity on tumor targeting. Mol Cancer Ther 8: 2861–2871. 19825804 10.1158/1535-7163.MCT-09-0195 PMC4078872

[38] Pearson JW, FitzGerald DJP, Willingham MC, Wiltrout RH, Pastan I, et al (1989) Chemoimmunotoxin Therapy against a Human Colon Tumor (HT-29) Xenografted into Nude Mice. Cancer Res 49: 3562–3567. 2499420

[39] Yang DJ, Azhdarina A, Wu P, Yu DF, Tansey W, et al (2001) In vivo and in vitro measurement of apoptosis in breast cancer cells using ^99m^Tc-EC-annexin V. Cancer Biother Radiopharm. 16: 73–83. 10.1089/108497801750096087 11279800

[40] Mochizuki T, Kuge Y, Zhao S, Tsukamoto E, Hosokawa M, et al (2003) Detection of tumor response following a single dose of chemotherapy with ^99m^Tc-annexin V. J Nucl Med. 44: 92–97. 12515881

[41] Schroit AJ, Madsen JW, Tanak Y (1985) In Vivo Recognition and Clearance of Red Blood Cells Containing Phosphatidylserine in Their Plasma Membranes. J Biol Chem 260: 5131–5138. 3988747

[42] Tait JF, Smith C, Blankenberg FG (2005) Structural requirements for in vivo detection of cell death with 99mTc-annexin V. J Nucl Med. 46: 807–815. PMC1201384 15872355

[43] Robidoux A, Buzdur AU, Quinaux E, Jacobs S, Rastogi P, et al (2010) A Phase II Neoadjuvant Trial of Sequential Nanoparticle Albumin-Bound Paclitaxel Followed by 5-Fluorouracil/Epirubicin/Cyclophosphamide in Locally Advanced Breast Cancer. Clin Breast Cancer 10: 81–86. 20133263 10.3816/CBC.2010.n.011

[44] Fang J, Nakamura H, Maeda H (2011) The EPR effect: Unique features of tumor blood vessels for drug delivery, factors involved, and limitations and augmentation of the effect. Adv Drug Deliv Rev. 18: 136–151. 10.1016/j.addr.2010.04.009 20441782

